# Thrombosis after Typhoid Fever

**Published:** 1902-12-06

**Authors:** 


### THROMBOSIS AFTER TYPHOID FEVER.

Clinicians often hesitate to accept the results of laboratory investigations as guides in practice, but the very interesting investigations by Professor Wright and Dr. Knapf, in regard to the causation of thrombosis in connection with typhoid fever, which were communicated to the last meeting of the Royal Medical and Chirurgical Society, are so strikingly in accord with certain facts, well known to clinical observers, that they are deserving of the most careful consideration, throwing light as they possibly may upon a long series of hitherto unexplained phenomena. These gentlemen set themselves to ascertain the coagulation time of the blood and its contents in lime salts in patients in the acute stage of typhoid fever, in convalescence from that disease, and in normal persons. The results of the blood examinations undertaken upon typhoid fever patients showed a general diminution of blood coagulability during the acute stage, while in convalescents there was a marked increase of coagulability. In eight cases which were examined both during the course of the pyrexia and after the return of the temperature to the normal, it was found that the coagulation time was on the average four and a half times shorter in the convalescent stage than during the course of the fever. In four of these cases the blood coagulation was abnormally rapid, and, in each of these, femoral thrombosis had taken place—these being the only cases among those examined in which it had occurred. Turning now to the estimation of the calcium salts in the blood, it was found that the average proportion of lime salts was in the case of convalescents from typhoid fever about twice that in normal blood. The presence of these two mutually related conditions — namely, increased amount of lime salts in the blood and increased coagulability — during the convalescent stages of typhoid fever, would seem then to afford a probable explanation of the well-known tendency to the occurrence of thrombosis at that stage of the disease, and here we come to the practical application of the theory. For whence comes this excess of lime salts, upon which depends the increased coagulability to which the tendency to thrombosis is due? The answer to this important question, according to the authors of the paper, is that this excess of lime salts is probably derived from the milk which for the most part constituted the exclusive dietary of the patient. Cow's milk contains 1 part in 600 of CaO, as compared with 1 part in 800 contained in lime water. This explanation receives support from several other observations, for if the milk dietary of the typhoid fever patient is the key to the frequency of thrombosis in the period of convalescence, a clue has probably been obtained to the resolution of other problems, as for example the frequently beneficial action of a milk diet on "serious hæmorrhage" from the kidney, and the comparative rarity of thrombosis after acute fevers, such as Malta fever, where a milk dietary is not imposed upon the patient.

The remedial measure would seem to be the exhibition of citric acid by which the excess of lime salts and the consequent excessive coagulability of the blood may be diminished. The same treatment, initiated as soon as the danger of intestinal hæmorrhage has passed away, would be appropriate for the prophylaxis of thrombosis in typhoid fever; or as an alternative a partial decalcification of the milk might be undertaken by adding to it citrate of soda in the proportion of from 20 to 40 grains to 1 pint. Lime salts probably are not the sole cause of thrombosis, but according to Professor Wright they are so important a factor that in the presence of a high percentage of lime salts a very slight change in the other constituents of the blood may determine a thrombosis; while in typhoid fever the excess of lime salts is the principal factor in the production of this accident.

